# Evaluation of Hungarian monitoring results and source localization of the ^106^Ru release in the fall of 2017

**DOI:** 10.1007/s10661-019-7567-0

**Published:** 2019-06-12

**Authors:** Dorottya Jakab, Tünde Ádámné Sió, Gáborné Endrődi, Zsolt Homoki, Sándor Kapitány, András Kocsonya, Júlia Kövendiné Kónyi, András Lencsés, László Manga, Annamária Pántya, Tamás Pázmándi, Krisztián Radó, Péter Rell, Péter Turza, Péter Zagyvai

**Affiliations:** 1 Hungarian Academy of Sciences Centre for Energy Research, 29-33 Konkoly-Thege Miklós street, Budapest, H-1121 Hungary; 2 National Food Chain Safety Office, 13-15 Fogoly street, Budapest, H-1182 Hungary; 3 National Research Institute for Radiobiology and Radiohygiene, 5 Anna street, Budapest, H-1221 Hungary; 4 Hungarian Atomic Energy Authority, 4 Fényes Adolf street, Budapest, H-1036 Hungary; 5 MVM Paks Nuclear Power Plant Ltd., Paks, H-7030 Hungary; 6 Public Limited Company for Radioactive Waste Management, Bátaapáti, H-7164 Hungary; 7 Public Limited Company for Radioactive Waste Management, Püspökszilágy, H-2166 Hungary

**Keywords:** Ru-106, Environmental radiological monitoring, Dose calculation, Source localization, Backward trajectory simulations

## Abstract

Anthropogenic ^106^Ru has been detected in the environment from late September to early October 2017 by several European environmental radiological monitoring networks. The paper presents the comprehensive evaluation of Hungarian monitoring results related to the occurrence of ^106^Ru in various environmental compartments (airborne particulates, deposition, plants, and terrestrial indicators), which was implemented to determine the temporal and spatial variation of the contaminant on a national scale and also to verify the findings based on the data arising from environmental monitoring at a local scale in Budapest. Difficulties in direct comparison of the diverse reported data were also considered; results arising from varied sampling periods were corrected with account taken of the relation between the sampling duration and 4-day-long plume residence (estimation based on the daily monitoring of air and backward trajectory analysis). Integrated analysis of air and deposition measurements and meteorological data was also performed; the deposition processes were investigated by establishing the correlations of activity concentrations measured in the atmosphere and in the deposition samples. In order to study the temporal distribution and spatial localization of the ^106^Ru contamination and to interpret the measurements at ground level, backward trajectory analysis was performed with HYSPLIT model. The backward trajectory simulations suggested that the release had probably occurred during the last week of September 2017 from the geographical area between Volga and the Urals. In addition, assessment of the doses due to the ^106^Ru release was implemented considering external exposure from cloudshine and groundshine and internal exposure via inhalation.

## Introduction

In a short period from late September to early October of 2017, several European networks involved in environmental radiological monitoring reported the detection of ^106^Ru isotope in environmental samples. Correspondingly to the European observations, ^106^Ru was also detectable by the stations of the Hungarian environmental radiological monitoring networks. Following the first registered detections, the European monitoring networks were formally requested by the Incident and Emergency Centre (IEC) of the International Atomic Energy Agency (IAEA) (IAEA 2017a) to provide measurement results related to the appearance of ^106^Ru in the environment in order to collect and analyze the measurement data and—if necessary—to recommend the implementation of public protective actions. The reported ^106^Ru activity concentration values higher than the minimum detectable activity (MDA) concentrations in the atmosphere over Europe varied in the range of 0.8 μBq·m^−3^–145 mBq·m^−3^ (IAEA 2017b; IRSN 2018); however, it must be noted that the air sampling duration at the data supply monitoring stations widely varied.

Large-scale emission of gaseous ruthenium oxides from the nuclear fuel can occur in high temperature and oxidizing atmosphere through volatilization, in which ruthenium oxides (RuO, RuO_2_, RuO_3_, and RuO_4_) are generated from the metallic state of ruthenium. However, during the examined event, ^106^Ru was detected solely in the environment, not as a minor component in a mixture of radionuclides of artificial origin, like following the Chernobyl nuclear power plant accident in 1986 (Steinhauser et al. 2014). The absence of other fission products excluded the assumption of a potential release from a nuclear reactor, which would have resulted the occurrence of other artificially produced radionuclides. The release of volatile ruthenium oxides (RuO_4_ is the most volatile of them) can also occur through certain technical phases and processes of nuclear fuel reprocessing (dissolution process of spent fuel in nitric acid, denitrification, evaporation, and vitrification of highly active waste concentrate) (Kleykamp 1988).

^106^Ru is commonly utilized for medical application, in particular for ocular brachytherapy for small- to medium-sized uveal melanoma (Stöckel et al. 2018) and retinoblastoma treatments (Schueler et al. 2006). The reported air concentrations were unlikely caused by medical treatment due to the small activity level (typical activity is up to 50 MBq (IAEA 2004)) of the ocular ^106^Ru brachytherapy sources.

Since detection of ^106^Ru in various environmental compartments happens to be a scarce phenomenon, studies on the direct measurements on the appearance of ^106^Ru contamination in the environment, the dispersion, and the radiological consequences of the release attain significance.

The objective of the evaluation of the available Hungarian environmental monitoring results was to analyze the concentration levels of ^106^Ru activity on a national scale and assess the dose consequences to contribute to the information related to national radiation conditions and radioactive contamination. The temporal and spatial behavior of the contaminated plume was also studied with backward trajectory analysis. Simulations were used to describe the atmospheric transport and dispersion of air parcels to interpret measurements on ground level, in order to locate the potential release region of the contaminated air masses as well as to estimate the travel time from the release at the source location and the residence time of the contaminated plume over the territory of Hungary. Additionally, the occurred incident causing unplanned discharge of ^106^Ru to the environment provided an opportunity to assess the data supply procedure related to the notification obligations.

## Materials and methods

### Evaluation of the monitoring of ^106^Ru in environmental media

In this study, occurrence of ^106^Ru in various environmental media was investigated, based on total beta counting and gamma spectrometric analysis of environmental samples (airborne particulates, wet and dry deposition, grass, other plants) collected at different locations in Hungary by the National Environmental Radiological Monitoring System (NERMS). According to the Government Decree 489/2015 (XII. 30.) (Hungarian Government 2015), NERMS has the obligation to acquire, analyze, register, and evaluate results related to environmental radiation measurements within the territory of Hungary and has to contribute to the fulfillment of the national and international notification and information obligations related to national radiation conditions and radioactive contamination. Following the first reported ^106^Ru detections in national and European level, NERMS had the obligation to collect the measurement data from the environmental radiological monitoring stations and report to the competent authorities of the IAEA (IAEA 2017a).

According to the regulation, NERMS members as public administration organizations (authorities), special facilities (nuclear power plant, research reactors, spent fuel interim storage facility, radioactive waste repository, radioactive waste treatment and disposal facility, research centers) and other institutions are obliged to participate in the activity of the NERMS. The distribution of the monitoring network locations throughout the country provides representative geographical coverage of the Hungarian territory. The monitoring programs of NERMS members correspond to the obligatory measured samples and quantities specified in the pertinent governmental decree (Hungarian Government 2015), which requirements are in compliance with the European Commission recommendations determined in 2000/473/Euratom (Commission of the European Communities 2000). According to the regulatory requirements, measurement of airborne particulates, ambient gamma dose rates, surface water, drinking water, milk, and mixed foodstuff is obligatory on a routine basis.

Key technical considerations regarding the measurement of low-activity, pure β-emitter ^106^Ru had been discussed by Jakab et al. (2018) and by Hult and Lutter (2017). In the following, general review will be given on the Hungarian monitoring of environmental compartments in relation to the ^106^Ru detections.

The activity concentration of aerosol-bond ^106^Ru in ground-level air was determined based on the continuous sampling of aerosol particulates. The air samplings were typically performed on glass-fiber filters at a constant air flow rate for a definite time period. The air flow rate at the measurement stations varied between the range of 3 and 250 m^3^·h^−1^ depending on the flow rate of the operated air pumps. Sampling time varied from 1-day- to 2-week-long intervals at different measurement locations depending on the actual decision between routine or increased sampling frequency. Extended sampling periods were generally required at monitoring stations equipped with low-flow-rate air samplers to ensure sufficient detection limits. The air filters were generally changed in the mornings on a routine basis. The continuously collected air samples were subjected to routine methods of radionuclide analysis, typically total beta counting with proportional counters and gamma spectrometry. Although the total beta counting method is not able to result in specific activities for individual β-emitters present in aerosol air filters, after all, it provided a rapid screening to indicate the elevated range of radiation levels in sampled ground-level air due to the ^106^Ru contamination mostly by means of the high-energy beta decay of its short-lived descendant ^106^Rh. Gamma spectrometry provided a tool to confirm the ^106^Ru presence in environmental samples and nuclide-specific determination of ^106^Ru also through ^106^Rh, without the necessity of chemical separation or any specific pretreatment of the measured air filters. In the majority of cases, high-purity germanium (HPGe) detectors with relative efficiencies of 20–60% were used for the identification and quantification of ^106^Ru/^106^Rh. MDA of ^106^Ru in air samples varied on a wide range between 0.01 and 1.2 mBq·m^−3^ due to the different volumes of sampled air, the diverse relative detection efficiencies of the used detectors, and variant counting times.

The majority of the reported Hungarian air measurement results were based on aerosol air filters sampled over a 7-day-long period. However, as it was noted, the duration of air sampling varied in time at certain measurement stations. Because of the varied sampling durations, the direct comparative analysis of the measured values was difficult. At those measurement points, where the sampling period exceeded the duration of the residence time estimated on the available daily monitoring results, the integration over the whole sampling interval would have resulted in an underestimation of the expected air concentration. In order to make the results comparable and to provide reliable estimation, the relation of the length of sampling interval to the residence time of the contaminant had to be taken into consideration. Measurement results therefore were corrected by weighting with the ratio of the sampling duration to the estimated average residence time of ^106^Ru in the territory of Hungary at a representative measurement point. This definition implies the assumption of an identical residence time for each sampling location, thus slightly increasing the overall uncertainty of the final result but providing input values for an overall dose assessment for the country.

Deposited activity of ^106^Ru radionuclide was also monitored additionally to the aerosol measurements. Primarily combined sampling of wet and dry deposition (fallout) was carried out with weekly to monthly sampling frequencies. The continuous sampling was complemented with subsequent radionuclide analysis (gamma spectrometry), following the pretreatment of the sample (in particular concentrating with evaporation). The measured activities were related to the collecting surface of the sampling vessels, which varied from 0.15 to 1.0 m^2^. Because of its lower sampling frequency, measurement of deposition samples was unable to detect the contamination rapidly and to monitor short-term variation of radiation levels. Nevertheless, the monitoring of the deposited activity enabled to measure the accumulated coverage of radionuclides on the ground and to follow the dispersion of discharged radionuclides in the environment due to the meteorological conditions.

Plant and terrestrial indicator (in particular grass) measurements were typically implemented promptly, so the routinely used drying or ashing processing steps were skipped and the sample pretreatment was restricted to compaction. It was all the more feasible as the vast majority of ^106^Ru present in these samples is assumed to occur due to surface deposition and not due to root uptake. At several stations, freshly harvested plant and grass samples were analyzed, so the measured activities were related to the fresh weight of the samples, which limited the sensitivity (i.e., MDA) of plant measurements.

### Dose estimation calculations

Three possible pathways of exposure were considered for dose estimation: (a) cloudshine dose due to gamma radiation from the passing radioactive plume, (b) groundshine dose due to radioactivity deposited on ground surface, and (c) inhalation dose due to particulates. The effective external and inhalation doses for reference population subgroups of 3-month-old infants; 1-, 5-, 10-, and 15-year-old children; and adults were derived on the basis of environmental monitoring data in compliance with the recommendations specified in the pertinent ICRP documents.

Dose assessment was performed on the basis of national average air and deposition monitoring data considering conservative assumptions (continuous exposure during the radioactive plume residence, whole fraction time spent in the radiation field in absence of shielding). Effective dose coefficients for the radioactive plume and for the deposition on the ground surface were obtained from the Federal Guidance Report No. 12 (Eckerman and Ryman 1993). The age-dependent effective dose coefficients for inhalation of ^106^Ru radionuclide and the inhalation rates were applied from the ICRP Publication 119 (Eckerman et al. 2012). Inhalation rates were determined from the default daily air intakes averaged over a 24-h period for each population subgroups. The daily inhalation was taken to be 2.86, 5.16, 8.72, 15.3, 20.1, and 22.2 m^3^ for 3-month-old infants; 1-, 5-, 10-, and 15-year-old children; and adults, respectively. The used procedure provides a rather conservative dose assessment as it assumes that the air concentration does not depend on whether the persons stay indoors or outdoors.

### Backward trajectory simulations

The temporal and spatial behavior of the contaminated plume was studied with backward trajectory simulations. Inverse atmospheric dispersion modeling could be used in a variety of simulations describing the transport and dispersion of air parcels to interpret the ^106^Ru measurements and to locate the possible origin region of the contaminated air masses, as well as to estimate the travel time from the release at the source location and residence time of the contaminated plume over a certain area. The simulations of the trajectories were performed with the Hybrid Single-Particle Lagrangian Integrated Trajectory model (HYSPLIT) developed by the US National Oceanic and Atmospheric Administration (NOAA), which is a hybrid model combining the Lagrangian approach and the Eulerian methodology (see detailed description in Stein et al. 2015). The required meteorological input data were obtained by the Global Data Assimilation System (GDAS). The publicly accessible meteorological dataset was obtained from the 3-, 6-, and 9-h forecasts as model output based on the 3-hourly archive data came from NCEP’s GDAS.

Since the ^106^Ru activity concentration dispersion pattern in Hungary showed low temporal and spatial variation, the backward trajectories were calculated from one fixed representative receptor point located in Central Hungary (at 47.151900 latitude and 18.867300 longitude coordinates) to cover the territory of the Hungarian region. A total of 12 simulations were performed backward-in-time from 12 UTC 4 October to 00 UTC 27 September. The total run time, which corresponds to the trajectory duration, was 120 h, which provided 5-day-long data of the atmospheric transport of air masses. At each simulation, three vertical levels of trajectory were calculated at 50, 75, and 100 m height above the ground-level receptor with respect to the meteorological data in each pressure level.

## Results and discussion

### Measurement results of Hungarian monitoring of ^106^Ru

Measurement results from 22 monitoring stations were evaluated based on the available data provided by the monitoring data supply centers of NERMS. ^106^Ru was detectable in environmentally monitored constituents, as air (aerosol air filters), deposition, plants, and terrestrial indicators (in particular grass).

### Results of total beta measurements of aerosol air filters

The daily monitoring of aerosol air filters to detect airborne particulate ^106^Ru enabled the determination of short-term variation of ^106^Ru activity concentration in ground-level air as it was described by Jakab et al. (2018). Evaluation of the national daily monitoring results enabled the estimation of the date of ^106^Ru occurrence and residence time of the radioactive plume in the atmosphere over Hungary. The comparative analysis of the available national daily monitoring results also allowed the verification of the findings based on the daily measurements at the KFKI Campus in Budapest.

**Fig. 1:**
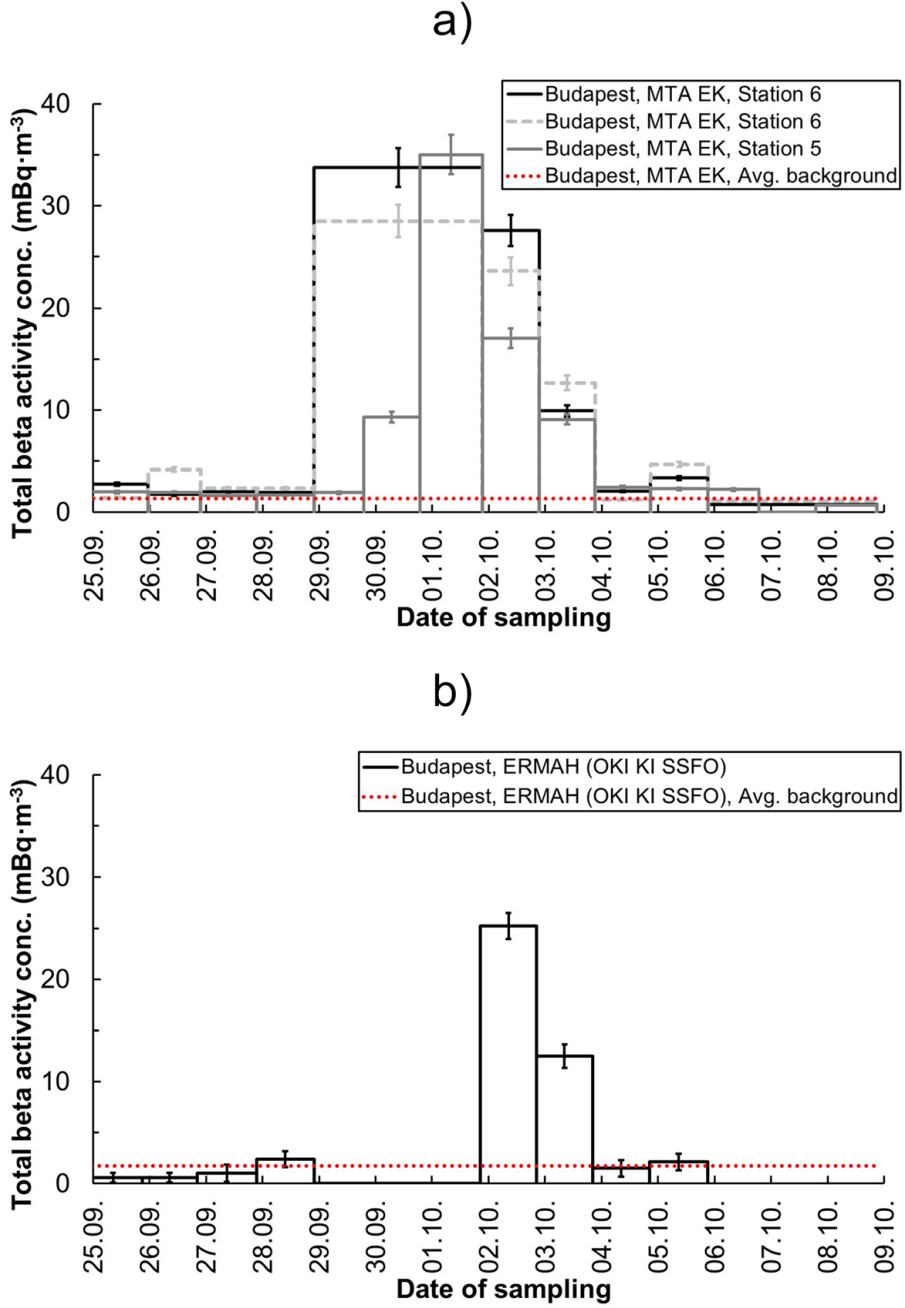
**a** Temporal variation of total beta activity concentration of aerosol air filters from 25 September to 9 October 2017, reported by MTA EK. Sampling was performed with 100–150 m^3^·day^−1^ flow rate air samplers. At stations 2 and 6 daily sampling was executed, except the weekends, where averaged values integrated over 3 days (from Friday morning to Monday morning) were presented. At station 5, sampling was performed continuously on a daily basis, including the weekends. **b** Temporal variation of total beta activity concentration of aerosol air filters from 25 September to 9 October 2017, reported by the monitoring data supply center ERMAH (OKI KI SSFO). At the measurement site daily sampling was performed, except the weekends, from which measurement results were not available

The available daily total beta activity concentrations (see Fig. 1) provided a rapid indication of the contamination. The first detections of increased level of total beta activity due to the appearance of ^106^Ru in the ground-level air were observed in the air filters sampled between 30 September and 1 October. The arrival date of the radioactive plume agrees with the first detections in Stockholm, Sweden (Ramebäck et al. 2018), and corresponds to the reported observations in Austria and the Czech Republic, based on the results published by IRSN (2018). The presence and contribution of ^106^Ru to the elevated total beta activity concentration was proven by subsequent nuclide-specific gamma spectrometry analysis of the filters. The maximal reported atmospheric total (gross) beta activity value (35.0 ± 2.0 mBq·m^−3^) was observed in Budapest by the Environmental Protection Service of MTA EK, between the mornings of 1 October and 2 October. This sample was evaluated according to the routine procedure of the service; thus, contribution of short-lived radon progeny was eliminated. The results obtained from daily sampling later than 4 October showed a steady decrease and the elevated level of total beta activity decreased to the range of the average background radiation level originated from the presence of natural beta-emitter components (1.4 ± 0.8 and 1.8 ± 0.4 mBq·m^−3^ in the territory of MTA EK and the measurement station of OKI KI SSFO, respectively) as the subsequent measurements generally did not determine increased quantity of total beta activity (as it can be seen in Fig. 1). It can be observed that the ^106^Ru activity concentration in ground-level air decreased gradually because of the removal from the plume due to deposition mechanisms in addition to the passing of the plume. The daily variation of total beta activity levels indicated a maximum of 4-day-long residence time correspondingly to the daily monitoring results observed in Budapest by the Environmental Protection Service of MTA EK (Jakab et al. 2018). According to the national monitoring data, it was verified that the ^106^Ru contamination was present over the territory of Hungary from 30 September until the morning of 4 October.

### Results of gamma spectrometric measurements of aerosol air filters

Table 1 gives a general overview of the measured ^106^Ru activity concentrations in the atmosphere over Hungary during the given sampling intervals.

**Table 1:** Overview of the ^106^Ru measurement results in aerosol air filters reported by the monitoring data supply centers of NERMS

Measurement site, monitoring data supply center	Sample type	Sampling period	^106^Ru activity concentration (mBq·m^−3^)
Bátaapáti, RHK Kft.	Aerosol air filter	25.09–09.10	4.5 ± 1.4 (5)^a^
Budapest, MTA EK	Aerosol air filter	25.09–02.10	10.7 ± 0.6^b^
Budapest, MTA EK	Aerosol air filter	02.10–09.10	3.9 ± 0.2^b^
Budapest, MTA EK	Aerosol air filter	09.10–16.10	< 0.2^b,^^c^
Budapest, MTA EK	Aerosol air filter	02.10–03.10	24.1 ± 1.7
Budapest, MTA EK	Aerosol air filter	03.10–04.10	11.9 ± 0.9
Budapest, MTA EK	Aerosol air filter	04.10–05.10	< 1.2^c^
Budapest, NÉBIH	Aerosol air filter	25.09–02.10	14.7 ± 0.3
Budapest, NÉBIH	Aerosol air filter	02.10–09.10	5.6 ± 0.1 (2)^a^
Budapest, NÉBIH	Aerosol air filter	09.10–16.10	< 0.01^c^
Budapest, ERMAH (reference station)	Aerosol air filter	25.09–02.10	10.6 ± 0.5
Budapest, ERMAH (reference station)	Aerosol air filter	02.10–09.10	4.7 ± 0.2
Budapest, ERMAH (reference station)	Aerosol air filter	09.10–16.10	< 0.01^c^
Budapest, ERMAH (OKI KI SSFO)	Aerosol air filter	25.09–02.10	12.0 ± 0.6
Budapest, ERMAH (OKI KI SSFO)	Aerosol air filter	02.10–05.10	9.2 ± 0.3
Budapest, ERMAH (OKI KI SSFO)	Aerosol air filter	05.10–09.10	0.3 ± 0.01
Budapest, BME NTI	Aerosol air filter	29.09–02.10	24.6 ± 2.0^d^
Budapest, BME NTI	Aerosol air filter	02.10–04.10	12.7 ± 1.0^d^
Győr, ERMAH	Aerosol air filter	26.09–03.10	14.3 ± 1.1
Miskolc, ERMAH	Aerosol air filter	29.09–05.10	14.6 ± 0.4
Miskolc, ERMAH	Aerosol air filter	29.09–09.10	10.0 ± 1.8
Paks, PA Zrt.	Aerosol air filter	25.09–02.10	13.5 ± 1.0 (9)^a^
Paks, PA Zrt.	Aerosol air filter	02.10–06.10	6.6 ± 0.7 (3)^a^
Paks, PA Zrt.	Aerosol air filter	02.10–09.10	4.0 ± 0.6 (6)^a^
Püspökszilágy, RHK Kft.	Aerosol air filter	02.10–05.10	8.7 ± 0.7^d^
Püspökszilágy, RHK Kft.	Aerosol air filter	05.10–09.10	2.4 ± 0.2^d^

^a^Average ^106^Ru activity concentration of simultaneous samplings over the same sampling period from the same area. The number of the averaged values, which were used to calculate arithmetic mean, is indicated in parenthesis

^b^Daily collected air filters obtained in one-week intervals were combined and measured collectively providing weekly average values over the sampling period

^c106^Ru activity concentration is given in < MDA format, when the measured value was below MDA for the given sample

^d^Uncertainty for the individual sample has not been determined; approximate uncertainty was calculated based on the average uncertainty typical for the gamma analysis of aerosol air filters

**Fig. 2:**
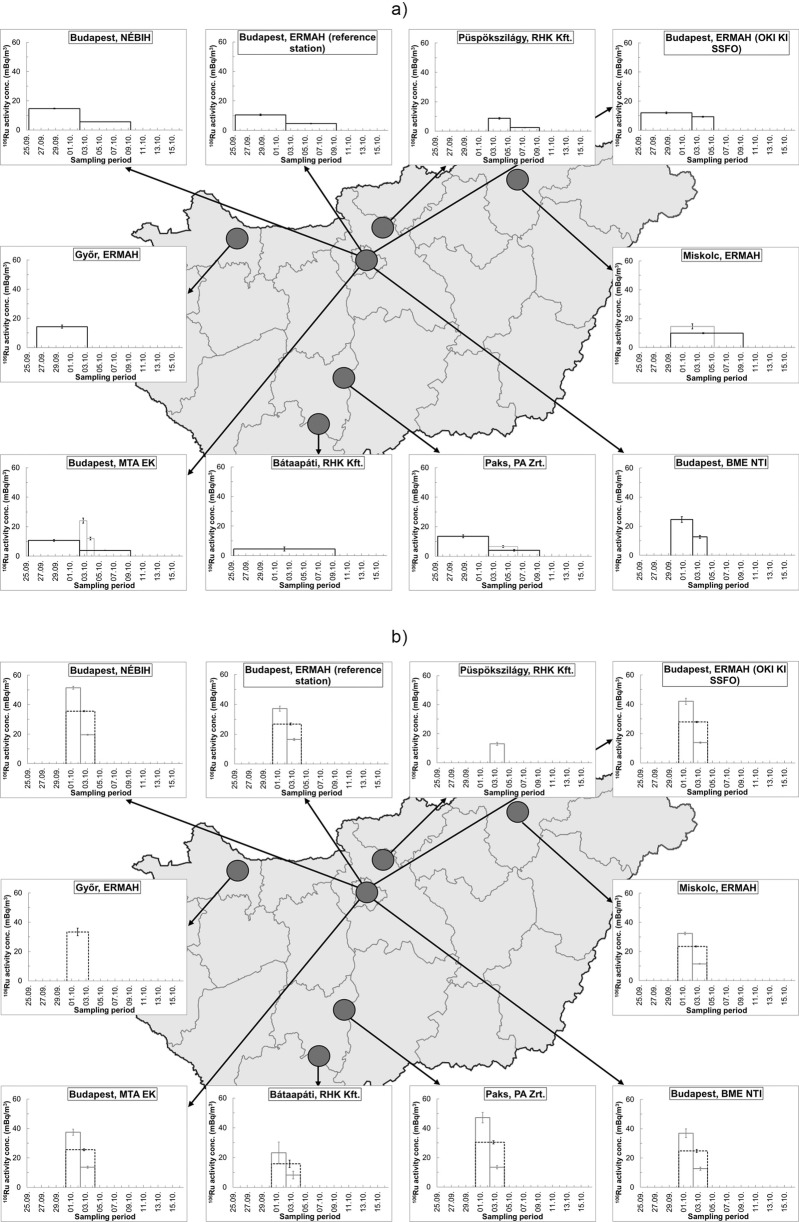
**a** Variation of ^106^Ru activity concentration in aerosol air filters as a function of time, obtained from the gamma spectrometry analysis of the sampled filters in Hungary over the time period of 25 September to 16 October 2017. The histogram columns correspond to the measured ^106^Ru activity concentrations over the sampling period. Where simultaneous sampling was performed, average values were calculated and visualized. Duplicated lines indicate the result of overlapping samplings. **b** Corrected ^106^Ru activity concentration in aerosol air filters for estimated residence time of ^106^Ru over the sampling interval. Continuous lines of histogram columns correspond to the corrected ^106^Ru activity concentrations for the period between 30 September and 2 October and for the interval of 2–4 October. Dashed lines correspond to the average corrected ^106^Ru activity concentrations integrated over the whole estimated plume residence

Figure 2a shows the ^106^Ru activity concentration in aerosol air filters obtained from the gamma spectrometry analysis, regarding the time period of the sampling from 25 September to 16 October 2017. The figure also visualizes the geographical locations of the measurement points. The histogram columns on the diagrams illustrate well the variant sampling durations, and the differences between initial and endpoints of the samplings.

Average value of ^106^Ru activity concentration in the ground-level atmosphere over Hungary was 10.6 ± 1.3 mBq·m^−3^ integrated over the whole 2-week-long period (25 September–9 October 2017), covering all cases of ^106^Ru detections. To ensure the comparability of the measurement results, correction of the values was executed by weighting with the ratio of the sampling duration to the estimated residence time of ^106^Ru over the sampling interval at each measurement point. The corrected values (see in Fig. 2b) gave an average activity concentration of 25.0 ± 2.0 mBq·m^−3^ over the 4-day-long estimated exposure time (from the morning of 30 September to the morning of 4 October) of ^106^Ru in the atmosphere over Hungary. Based on the corrected results, it can be concluded that the measurement results obtained by different monitoring data supply centers are in a good agreement. ^106^Ru activity concentration dispersion pattern in Hungary showed low temporal and spatial variation, resulting relatively uniform levels of ^106^Ru contamination in the country.

From the data reports of a few monitoring data supply centers, measurement results of simultaneously sampled air filters (i.e., samples collected over the same interval) were available from the same locations. Parallel measurement results enabled the validation of the sampling and measurement procedures as well as the refinement of the outcomes of the calculations. Based on the comparison of simultaneous samplings, it can be determined that air monitoring results observed in the same area over a given sampling interval show a good agreement. For the comparative analysis of parallel air sampling, in the following section, ^106^Ru activity concentrations observed in simultaneously sampled aerosol air filters in Paks will be taken as an example. In the vicinity of the Paks Nuclear Power Plant (NPP), an environmental radiological monitoring system is operated, in which aerosol particulates are sampled at 9 stations—located within a 2-km radius around Paks NPP—routinely on a weekly basis. Simultaneous sampling was performed in Paks at 9 stations between the interval of 25 September and 2 October (arithmetic mean, 13.5 mBq·m^−3^; standard deviation, 1.0 mBq·m^−3^), whereas parallel sampling was carried out from 2 October to 6 October at 3 stations (arithmetic mean, 6.6 mBq·m^−3^; standard deviation, 0.7 mBq·m^−3^) and simultaneous measurement results are available from the remaining 6 measurement stations from the period between 2 October to 9 October (arithmetic mean, 4.0 mBq·m^−3^; standard deviation, 0.6 mBq·m^−3^). The simultaneous results from the 9 measurement points and parallel results from the 3 and the remaining 6 measurement stations varied with 7.7%, 10.3%, and 14.4% relative standard deviation, respectively. These relative standard deviations (i.e., standard deviation related to the average value) in the air measurements can be interpreted as an acceptable variation considering the typical uncertainty level of environmental measurements.

In the case of the air filters sampled in Paks, the 7-day-long sampling period exceeded the duration of the ^106^Ru contamination presence, which could cause an underestimation of the air concentration, as the measured value would be integrated over the total sampling interval (i.e., the sample activity divided by the total sampled air volume of that week). The measurement results therefore were corrected similarly as the national values (by weighting with the ratio of the sampling interval length to the estimated residence time of the plume). The corrected values gave an average activity concentration of 49.4 ± 3.8 mBq·m^−3^ for the period between 30 September and 2 October, which value was referred to as the maximal ^106^Ru activity concentration in the atmosphere over Hungary by some reports (IAEA 2017b; IRSN 2018).

### Results of the deposition measurements

Table 2 shows the detected ^106^Ru activity concentrations in deposition samples.

**Table 2:** Overview of the ^106^Ru measurement results in deposition samples reported by the monitoring data supply centers of NERMS

Measurement site, monitoring data supply center	Sample type	Sampling period	^106^Ru activity concentration (Bq·m^−2^)
Budapest, MTA EK	Deposition	04.09–02.10	< 2.8^a^
Budapest, MTA EK	Deposition	25.09–02.10	< 2.1^a^
Budapest, MTA EK	Deposition	02.10–09.10	11.3 ± 2.2 (2)^b^
Budapest, MTA EK	Deposition	02.10–06.11	11.3 ± 1.5 (4)^b^
Budapest, MTA EK	Deposition	09.10–16.10	< 2.5^a^
Budapest, ERMAH (OKI KI SSFO)	Deposition	02.10–02.11	5.4 ± 0.8
Budapest, NÉBIH	Deposition	01.10–05.10	5.5 ± 0.8
Paks, PA Zrt.	Deposition	05.09–02.10	< 7.0^a^
Paks, PA Zrt.	Deposition	02.10–06.11	6.5 ± 1.0 (2)^b^
Szekszárd, NÉBIH	Deposition	01.10–09.10	10.4 ± 2.0
Szekszárd, NÉBIH	Deposition	01.10–31.10	8.6 ± 0.4

^a106^Ru activity concentration is given in < MDA format, when the measured value was below MDA for the given sample

^b^Average ^106^Ru activity concentration of simultaneous samplings over the same sampling period from the same area. The number of the averaged values is indicated in parenthesis

Based on the available meteorological datasets, a dry period lasted in the interval between 24 September and 2 October in Hungary. During this period, naturally occurring ^7^Be was detectable in deposition; this cosmogenic radionuclide is suitable for quality control as the average and the range of specific activities of ^7^Be are known for each measurement location. Under dry conditions, ^106^Ru from the passing contaminated air masses deposited on the surface via dry deposition mechanism (resulted mainly from the downward vertical fall of atmospheric aerosols onto the surface) in the first days of the plume residence. ^106^Ru activity concentrations in deposition sampled before 2 October were below the detection limits (typically 2.1 Bq·m^−2^) at each measurement point, which indicates low deposition rates and high remaining activity in the plume. According to the dry deposition model, the dry deposition velocity (*v*_*d*_) of ^106^Ru aerosols, defined as the ratio of the ^106^Ru activity concentration deposited on the ground surface (dry deposition rate, *D* < 2.1 Bq·m^−2^) to the time integral of the average corrected concentration of ^106^Ru activity in air from the morning of 30 September to the morning of 2 October (*C*_*air*_ = 1.8 Bq·h·m^−3^), would be lower than 3.2 × 10^−4^ m·s^−1^. Because of the particle size dependency of the dry deposition velocity, the low value of the dry deposition velocity compared to the default values used by operational atmospheric dispersion models (reference values generally lie in the range of 10^−3^ m· s^−1^) follows from the presence of particles with small aerodynamic diameter (≤ 1 μm).

**Fig. 3:**
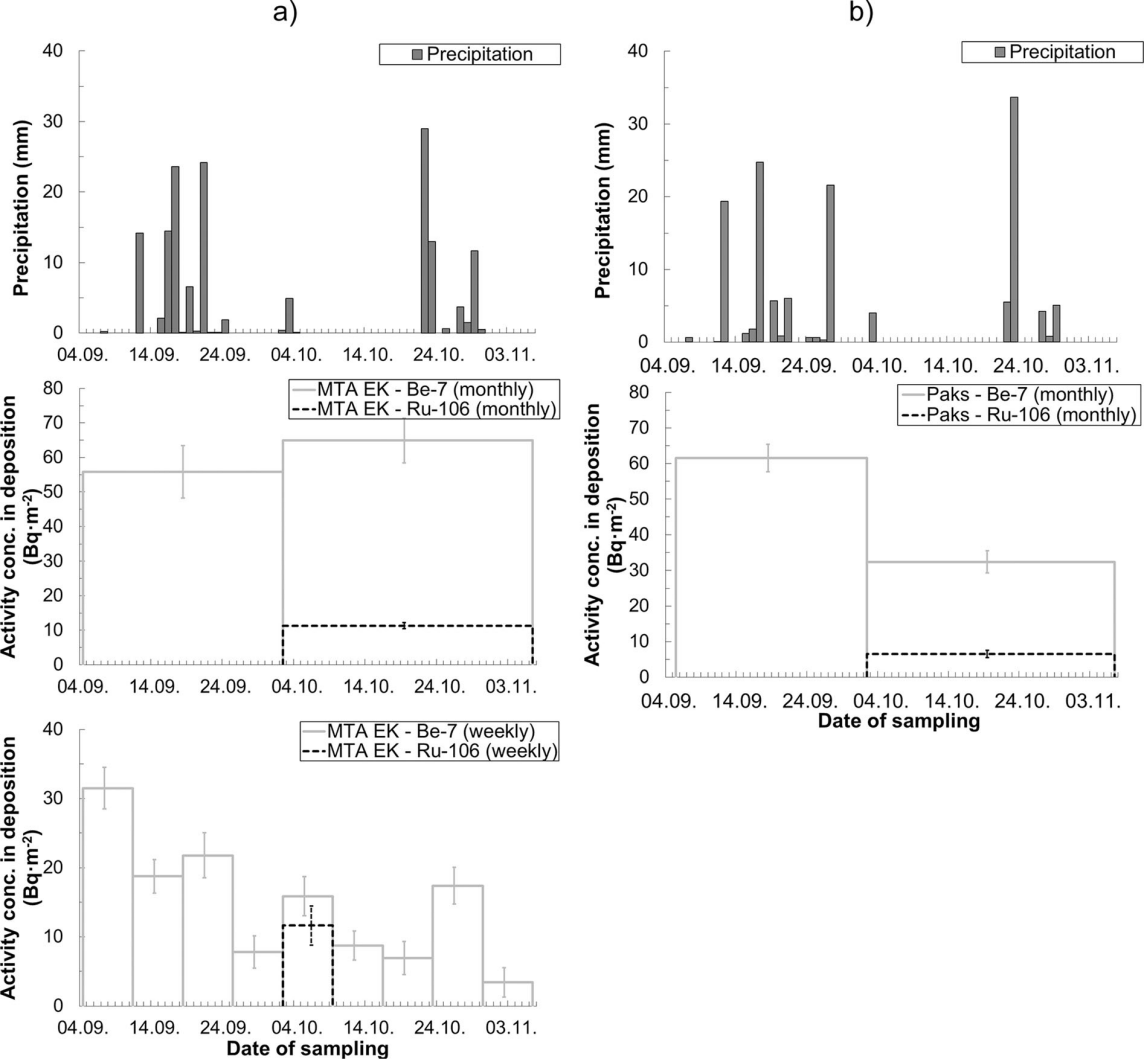
**a**, **b** Relation between the daily summation of precipitation amounts and deposited ^106^Ru activity concentrations. Panel **a** shows the weekly (visualized data for one station) and monthly measurements (visualized data for the weighted mean of four simultaneously collected samples taken between 2 October and 6 November) of MTA EK. Panel **b** shows the monthly measurements of Paks (visualized data for the weighted mean of two simultaneously collected samples for which reliable ^106^Ru data was available)

Integrated analysis of deposition measurement results and available meteorological data (see Fig. 3) indicated that deposition to the ground surface of ^106^Ru was strongly affected by the occurrence of precipitation. On a national scale, the first contiguous rainfall event with intensities exceeding the measurement resolution occurred on 3 October with about 1.2 mm·h^−1^ national average precipitation intensity related to the precipitation durations. Apart from some rare locally prevailing precipitation events, the spatial and temporal distribution of the precipitation in the territory of Hungary showed low variation during the plume residence. The evaluation of the Hungarian deposition values verifies the conclusion made on the basis of the deposition measurements by the Environmental Protection Service of MTA EK, namely that during the plume residence, wet deposition (due to the removal of radionuclides effected by precipitation mechanisms) was the dominant contributor to the deposition onto the surface as ^106^Ru was only detectable in deposition samples after 2 October (Jakab et al. 2018). This conclusion corresponds to the findings of Ramebäck et al. (2018) as well, where wet deposition as a consequence of the rainfall on early October was also identified as the major removal process. The Hungarian average ^106^Ru activity concentration in deposition was 8.4 ± 1.0 Bq·m^−2^.

Based on the weekly deposition sampling, trends of deposition in time could be determined. As it was formerly stated, ^106^Ru was only detectable between 2 and 9 October, in weekly collected deposition samples. ^106^Ru activity in deposition sampled after 9 October remained below the detection limit in spite of the subsequent heavy rainfalls in the period of 22–23 October and on 29 October. The undetectable ^106^Ru activity concentrations in deposition confirmed that the cloud had already passed by that period.

Parallel deposition measurement results showed a slightly higher variation than the simultaneous air measurements. The results of simultaneous monthly deposition measurements in the 4 measurement stations of MTA EK and parallel results from 2 measurement stations in Paks varied with 17.7% and 15.2% relative standard deviation, respectively. The differences between the deposition results observed in the same area over a given sampling interval can arise from the sampling uncertainty due to the natural variability of deposition, and the formation of local turbulent wakes generated by the ambient air flow in the area of the sampling equipment. Despite the variances between the parallel deposition measurements, the measured activities in deposition samples—correspondingly to the meteorological data—sufficiently indicated the temporal variation of the deposited ^106^Ru concentration and provided input data that could be used in atmospheric deposition models and dose assessments.

### Results of plant and terrestrial indicator measurements

Table 3 shows the detected ^106^Ru activity concentrations in plants and terrestrial indicators.

**Table 3:** Overview of the ^106^Ru measurement results in plants and terrestrial indicator samples reported by the monitoring data supply centers of NERMS

Measurement site, monitoring data supply center	Sample type	Sampling date	^106^Ru activity concentration (Bq·kg^−1^)
Bátaapáti, NÉBIH	Nettle	12.10	1.8 ± 0.4
Bokod, NÉBIH	Nettle	02.10	1.5 ± 0.4
Bokod, NÉBIH	Grass	02.10	10.7 ± 0.5
Budapest, MTA EK	Grass	09.10	< 3.2^a^
Budapest, MTA EK	Grass	16.10	< 4.6^a^
Budapest, NÉBIH	Grass	03.10	5.3 ± 1.9
Budapest, NÉBIH	Grass	09.10	2.9 ± 0.8
Budapest, NÉBIH	Grass	09.10	7.1 ± 0.8
Debrecen, NÉBIH	Grass	09.10	3.7 ± 0.5
Gyömrő, NÉBIH	Grass	05.10	1.6 ± 0.2
Gyömrő, NÉBIH	Sorrel	05.10	1.1 ± 0.2
Gyömrő, NÉBIH	Celandine	05.10	1.3 ± 0.2
Kaposvár, NÉBIH	Grass	09.10	2.4 ± 0.9
Kecskemét, NÉBIH	Grass	09.10	< 4.5^a^
Kisbér, NÉBIH	Grass	02.10	3.7 ± 0.5
Kisbér, NÉBIH	Nettle	02.10	1.4 ± 0.4
Maglód, NÉBIH	Sorrel	05.10	1.1 ± 0.2
Maglód, NÉBIH	Nettle	05.10	0.7 ± 0.3
Maglód, NÉBIH	Rosehip	05.10	0.2 ± 0.01
Mórágy, NÉBIH	Grass	12.10	1.5 ± 0.3
Miskolc, NÉBIH	Grass	09.10	< 6.0^a^
Paks, NÉBIH	Grass	05.10	2.3 ± 0.3
Súr, NÉBIH	Nettle	02.10	1.5 ± 0.4
Szekszárd, NÉBIH	Plant	09.10	1.8 ± 0.4
Szombathely, NÉBIH	Grass	09.10	< 3.6^a^
Veszprém, NÉBIH	Grass	09.10	< 4.0^a^

^a106^Ru activity concentration is given in < MDA format, when the measured value was below MDA for the given sample

**Fig. 4:**
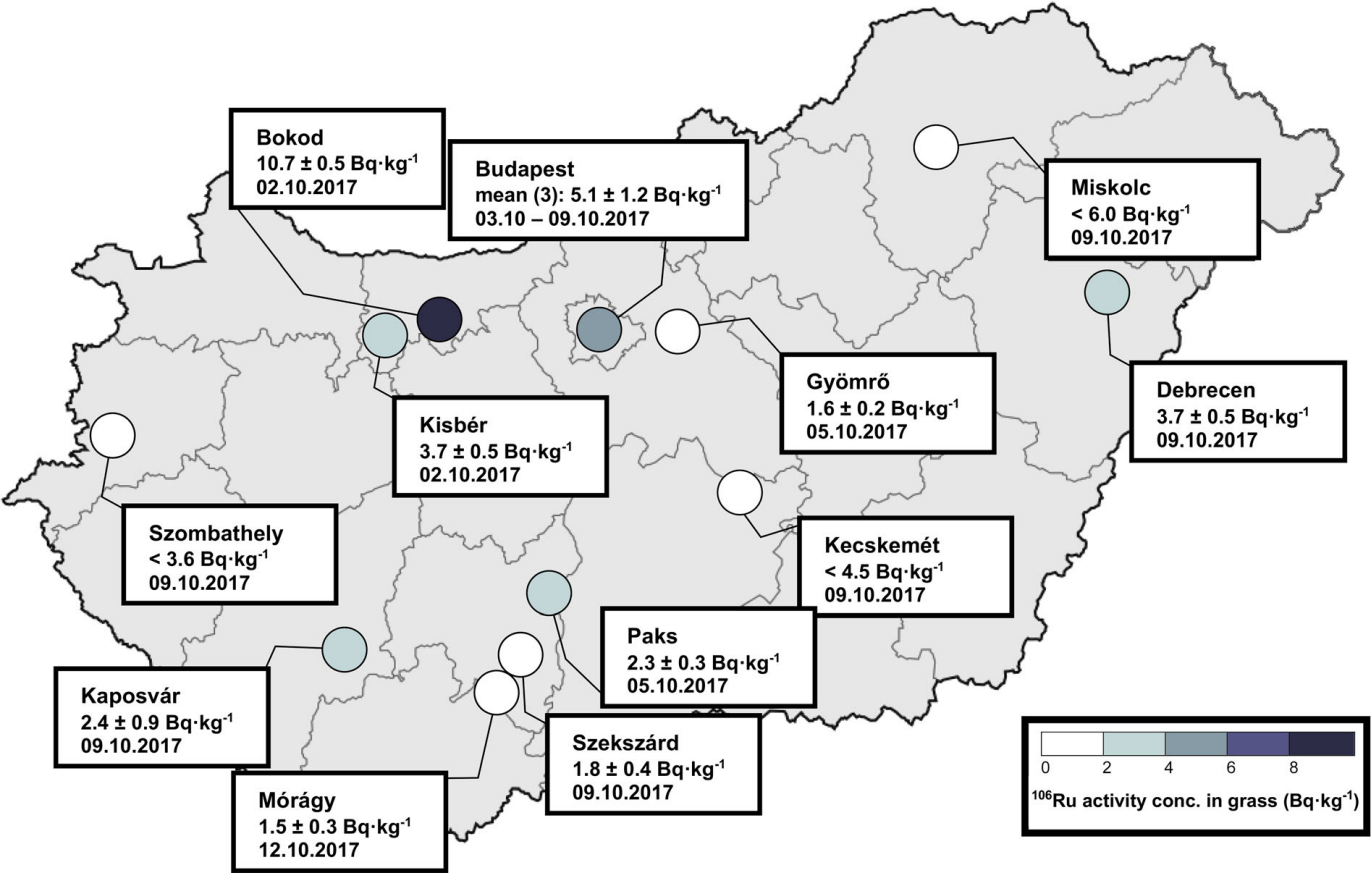
Spatial variation and range of ^106^Ru activity concentration in grass samples. As results of simultaneous samplings from the same area, arithmetic mean (in the figure, it corresponds to mean) of the ^106^Ru activity concentrations was given. The number of the averaged values is indicated in parenthesis

The vegetation sampling locations covered the territory of the Hungarian region sufficiently (see Fig. 4). The contamination of external plant and grass surfaces was also caused predominantly due to the wet deposition resulted from the rainfall event on 3 October. Grass samples were taken after 2 October yielded a maximum of 10.7 ± 0.5 Bq·kg^−1 106^Ru activity concentration related to the fresh weight.

The objective of the monitoring of plants could be the determination of the doses to members of the public from the ingestion of deposited radionuclides in foodstuffs. Plant surfaces are primarily contaminated by direct deposition of aerosol-bond radionuclides or by indirect contamination via resuspended radionuclides. Grass could be a direct pathway of the deposited radionuclides to animals by their rapid uptake of radionuclides.

### ^103^Ru detection in the atmosphere over Hungary

Predominantly, ^106^Ru have been detected solely in the atmosphere over Europe; nevertheless, simultaneous detection of ^103^Ru and ^106^Ru was reported by a few European environmental radiological monitoring networks. According to IRSN (2018), the 10.6 ± 1.5 mBq·m^−3^ average level of ^106^Ru was accompanied by 3.1 ± 0.5 μBq·m^−3^ average ^103^Ru activity concentration in air filters sampled in Sweden, Austria and the Czech Republic in the period between 25 September and 4 October. The ratio of average ^106^Ru and ^103^Ru activity concentrations corresponded to 3930. The cumulative fission product yield of shorter-lived ^103^Ru (*T*_*1/2*_ = 39.3 days; Bé et al. 2016) for ^235^U thermal and fast neutrons is (3.103 ± 0.084)% and (3.248 ± 0.042)%, whereas (0.410 ± 0.011)% and (0.469 ± 0.036)% of ^106^Ru, respectively. The cumulative fission product yield of ^103^Ru for ^239^Pu thermal and fast neutrons is (6.948 ± 0.083)% and (6.59 ± 0.16)%, whereas (4.188 ± 0.092)% and (4.13 ± 0.24)% of ^106^Ru, respectively, published by Koning et al. (2006). According to the fission yields, assuming that ^106^Ru and ^103^Ru were generated together in a reactor fuel of an appropriate light water reactor, the Ru species detected in the atmosphere over Europe were about 2 years old.

In aerosol filters sampled at the Hungarian monitoring stations ^103^Ru was not detected, even at the monitoring stations equipped with the highest-flow-rate (240–250 m^3^·h^−1^) air samplers. MDA of ^103^Ru with gamma spectrometry measurements was in the range of some tenths of microbecquerels per cubic meter, which exceeded the highest ^103^Ru activity concentration detected in Europe with one order of magnitude.

### Estimation of public exposure due to the ^106^Ru contamination

Based on the observed ^106^Ru activity levels in air, it can be concluded that 25.0 mBq·m^−3 106^Ru activity concentration in ground-level air would lead to 9.4 × 10^−4^ nSv·h^−1^ external gamma radiation dose rate, whereas ^106^Ru deposition of 8.4 Bq·m^−2^ would result in 6.4 × 10^−3^ nSv·h^−1^ ground radiation dose rate (calculated with the dose coefficients for air submersion and for exposure to contaminated ground surface given by Eckerman and Ryman (1993)). As the ambient gamma dose rate monitors have detection limits typically in the order of nanosievert per hour, it can be stated that ^106^Ru occurrence in the environment was not sufficient to cause a detectable increase of the ambient gamma dose rate.

Considering the inhalation dose of the examined population subgroups, the results varied with 21% relative standard deviation due to the age dependence of the dose coefficients and inhalation rates. The most exposed group of the population for internal exposure due to inhalation was the adult reference subgroup. The findings of the deposition model calculations based on the deposition measurements indicated the presence of particles with small aerodynamic diameter. Therefore, effective dose coefficients for inhalation of particulate aerosols with default activity median aerodynamic diameter (AMAD) of 1 μm were used. Internal dose from inhalation of airborne ^106^Ru radionuclide to the adult population group was estimated as 146 nSv, assuming constant inhalation of 25.0 mBq·m^−3^ activity concentration with exposure duration corresponding to the 4-day-long estimated residence time of ^106^Ru in Hungary. As a simplifying assumption, the resuspension mechanisms moved by the action of wind or by disturbances of the soil or external plant surfaces were not taken into account in the calculation of inhalation. External dose from cloudshine to the adult population group was 0.09 nSv, assuming a continuous exposure over the 4-day-long estimated residence time in 9.4 × 10^−4^ nSv·h^−1^ external gamma radiation dose rate as stated above. External dose from groundshine to the most exposed group in the first week following the initial deposition was 1.1 nSv with assumption that the radioactivity was removed from the ground surface only via radioactive decay. It must be noted that radioactive progeny was considered in external dose coefficients, the parent ^106^Ru and progeny ^106^Rh remain in secular equilibrium in the radioactive plume and in the subsequent deposition on the ground surface. External doses received by members of the examined subgroups were less variant because of the slight dependence of the dose coefficients on age.

The dose assessment showed that internal exposure due to the inhalation of contaminated air had paramount contribution to public exposure, whereas external pathways via exposure from cloudshine and groundshine had negligible contribution to the total effective dose which itself was also negligible. The total effective dose to the adult reference population subgroup in the estimated residence time of ^106^Ru was 147 nSv, which is 0.006% of the 2.4 × 10^6^ nSv worldwide average annual effective dose from natural sources of ionizing radiation (UNSCEAR 2000). This practically means that the received dose due to ^106^Ru exposure equals with the received dose from natural background radiation by spending about 2 h outdoors (as the population-weighted average absorbed dose rate in outdoors is 57 nGy·h^−1^ reported by UNSCEAR 2000).

### Trajectory analysis of the ^106^Ru contamination on European scale

**Fig. 5:**
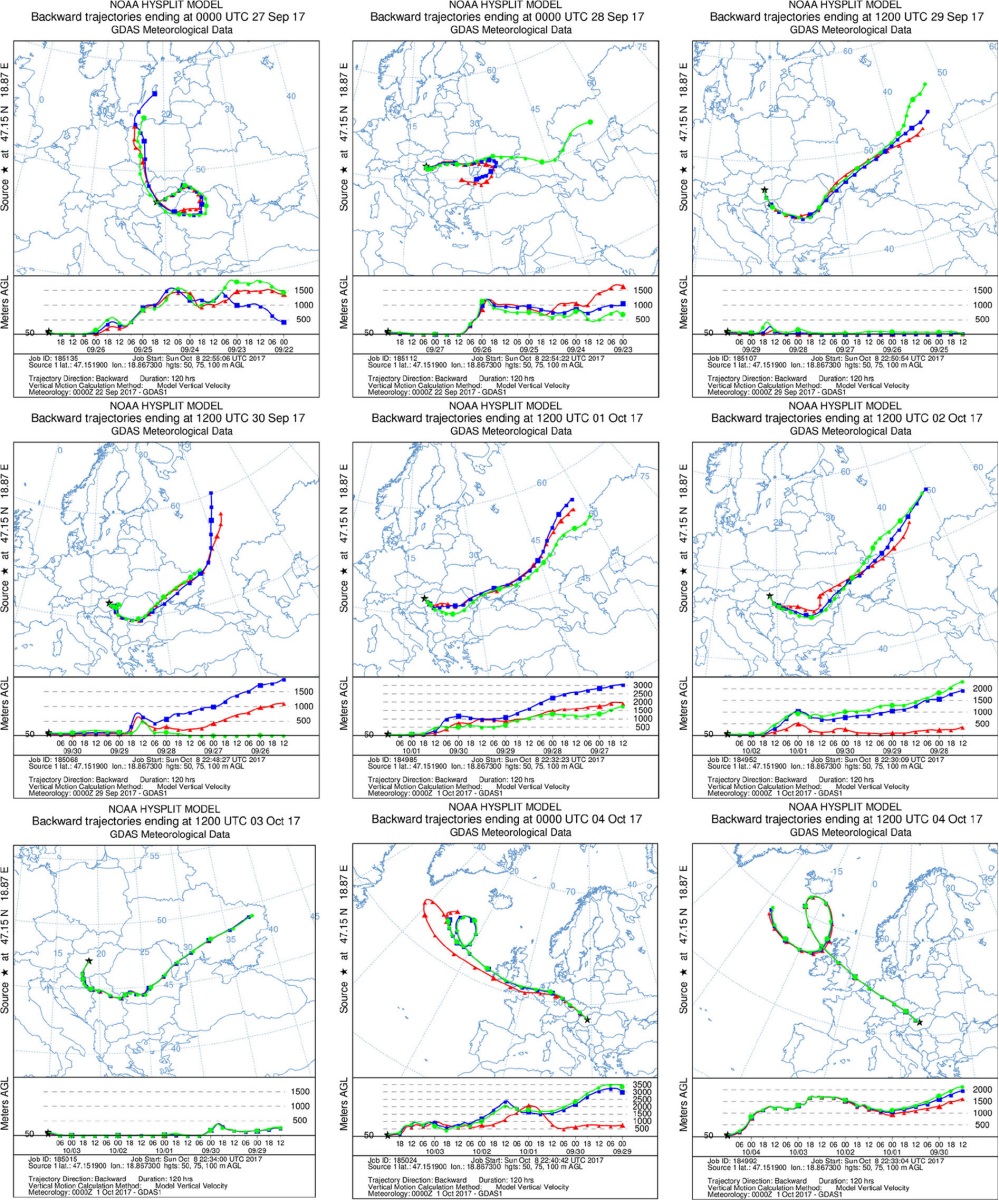
Evolution of the trajectories as a function of time based on the trajectory simulations backward-in-time from 12 UTC 4 October to 00 UTC 27 September. The receptor point (at 47.151900 latitude and 18.867300 longitude coordinates) was indicated with a black star on each map

Figure 5 represents the results of the trajectory analyses backward-in-time from 12 UTC 4 October to 00 UTC 27 September performed with HYSPLIT atmospheric transport and dispersion modeling system. The geographical location of the potential release zone was estimated with the assumption that the detected ^106^Ru was discharged to the atmosphere from a ground-level release point. Based on the executed simulations, it can be concluded that the most probable region of the release lies in the geographical area between the Volga and the Urals. The findings of the spatial localization are consistent with the outcomes of the simulations described by IRSN (2018) and Sørensen (2018).

The backward trajectory analysis result confirms the findings based on the assessment of the aerosol air filters that the radioactive cloud reached the territory of Hungary at the end of September 2017 from the determined release direction. As it can be seen on the trajectories inversely modeled from 4 October 2017, the wind direction changed in this time period, which eliminated further transport of potentially contaminated air masses from the region of origin resulting in consequent reduction of the environmental radiation levels of the atmosphere. The release, with regard to the backward trajectory simulations, could occur during the last week of September 2017.

Atmospheric dispersion model calculations are associated with uncertainties; these uncertain components (e.g., uncertainties of meteorological variables) have effect on the outcomes of the atmospheric dispersion modeling. In consequence of the above ascertainment, the overall uncertainty of the inverse trajectory modeling was relatively high; therefore, the more precise determination of the contaminant’s spatial localization was not feasible from the available data.

## Conclusions

Results of Hungarian radiation monitoring and environmental sampling stations were evaluated in terms of the occurrence of ^106^Ru in the environment over the period from late September to early October 2017. ^106^Ru activity concentrations in different environmental compartments (air, deposition, and plants as terrestrial indicators) were analyzed. Comparative analyses of the reported datasets, provided by the Hungarian monitoring data supply centers of NERMS, were implemented to determine the temporal and spatial variation of the radiation levels on a national scale and also to supplement and verify the outcomes of the evaluation based on the local data arising from the environmental monitoring in Budapest. The dispersion patterns of the ^106^Ru activity concentration in Hungary showed low temporal and spatial variation, which resulted in relatively uniform radiation levels at a national scale. The hypothesis on the maximum of 4-day-long residence time was verified based on the additional Hungarian daily monitoring data and the backward trajectory analysis. According to the investigation of the daily variation of Hungarian activity levels in ground-level air, the ^106^Ru contamination circulated over the territory of Hungary from 30 September until the morning of 4 October, correspondingly to the daily monitoring of air in Budapest. To enable direct comparability, the diverse available data were corrected with account taken of the relation between the sampling duration and the estimated 4-day-long residence time of the radioactive plume. With the correction of the raw national air monitoring data, average ^106^Ru activity concentration of 25.0 ± 2.0 mBq·m^−3^ has been calculated over the estimated residence time of ^106^Ru in ground-level air. Integrated analysis of deposition measurement results and available meteorological data verified that the deposition of ^106^Ru to the ground surface was dominantly influenced by the occurrence of rainfall episodes. Results indicated that wet deposition mechanism was the major contributor of the removal process from the plume to ground, which led to an average of 8.4 ± 1.0 Bq·m^−2^ deposition on the ground surface prior to the plume passage.

Backward trajectory analysis was used for the temporal distribution and spatial localization of the ^106^Ru contamination. The simulations were performed with HYSPLIT model on the basis of monitoring results and publicly accessible meteorological data. Based on the simulations, the geographical area between the Volga and the Urals was determined as the possible origin region. According to the backward trajectory simulation, the release occurred probably during the last week of September 2017.

Dose assessment was performed considering external exposure from cloudshine and groundshine and internal exposure via inhalation. The doses received by members of infant; 1-, 5-, 10-, and 15-year-old children; and adult reference population subgroups were estimated with conservative assumptions, on the basis of national average air and deposition monitoring data. According to the dose assessment, the estimated exposure was negligible; however, inhalation has been determined as the main exposure pathway, which contributed predominantly to the total effective dose (147 nSv on average) in short term. External pathways via exposure from cloudshine and groundshine had negligible contribution to the total effective dose which itself was also negligible.
